# Mechanism and Disease Association With a Ubiquitin Conjugating E2 Enzyme: UBE2L3

**DOI:** 10.3389/fimmu.2022.793610

**Published:** 2022-02-21

**Authors:** Xiaoxia Zhang, Chengdong Huo, Yating Liu, Ruiliang Su, Yang Zhao, Yumin Li

**Affiliations:** ^1^ Department of Ophthalmology, Lanzhou University Second Hospital, Lanzhou, China; ^2^ Key Laboratory of the Digestive System Tumors of Gansu Province, Lanzhou University Second Hospital, Lanzhou, China

**Keywords:** Ubiquitination, UBE2L3, NF-κB, p53, p62, cancer, immune diseases, Parkinson’s disease

## Abstract

Ubiquitin conjugating enzyme E2 is an important component of the post-translational protein ubiquitination pathway, which mediates the transfer of activated ubiquitin to substrate proteins. UBE2L3, also called UBcH7, is one of many E2 ubiquitin conjugating enzymes that participate in the ubiquitination of many substrate proteins and regulate many signaling pathways, such as the NF-κB, GSK3β/p65, and DSB repair pathways. Studies on UBE2L3 have found that it has an abnormal expression in many diseases, mainly immune diseases, tumors and Parkinson’s disease. It can also promote the occurrence and development of these diseases. Resultantly, UBE2L3 may become an important target for some diseases. Herein, we review the structure of UBE2L3, and its mechanism in diseases, as well as diseases related to UBE2L3 and discuss the related challenges.

## Introduction

Ubiquitination is an important posttranslational modification that regulates many cellular processes, including protein turnover and the stress response, the cell cycle, organelle synthesis, and the intracellular homeostasis maintenance (1). Thus, abnormities in ubiquitination can lead to the development of many serious diseases-related processes, such as tumors, neurodegeneration, immune diseases, and susceptibility to infections (2–4).

**Figure 1:**
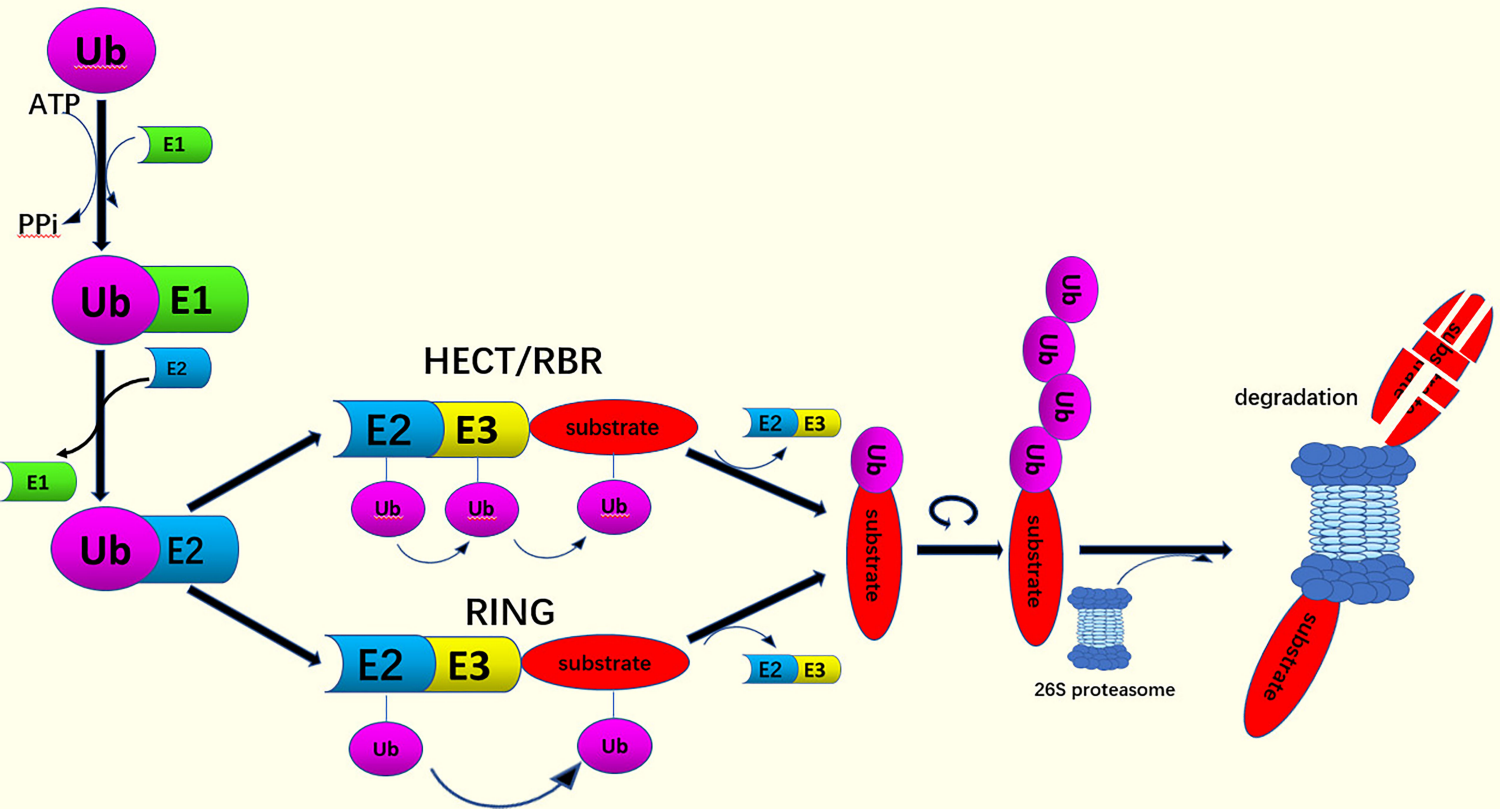
The process of ubiquitination. Ub is activated by E1 through an ATP-dependent step. The activated Ub attaches to the E2. The binding of Ub to target proteins can be catalyzed by HECT and RBR-type E3 ligases, or RING domain E3 ligases. This acts as a bridge between the activated E2 and the substrate indicating that the ubiquitin signals form polyubiquitin chains. Finally, the substrate is degraded by the 26S proteasome.

Small molecules of ubiquitin arrive at the substrate proteins to complete ubiquitination through a complex three-step enzyme cascade (5). Using ATP, the E1 ubiquitin-activating enzymes activate ubiquitin and form the ubiquitin-E1 complex. Ubiquitin, which is activated by adenylate, is transferred to a E2 ubiquitin-conjugating enzyme *via* a thioester bond to form the E2-ubiquitin complex (6). The E3 ubiquitin ligase enzyme then promotes the ubiquitin transfer from E2 to the substrate lysine to complete the ubiquitination process (6) (**Figure 1**). This process forms the functional site of ubiquitination- the isopeptide bond formed between the C- terminal glycine (Gly) of ubiquitin and the lysine on the substrate protein (6–8). Deubiquitin enzymes (DUBs) process ubiquitin precursors, edit the chain topologies, or cut ubiquitin from the substrates to terminate the signal transduction (9, 10).

Ubiquitin is an 8.9 kDa protein with 76 amino acids that is, attached covalently to the lysine residues on the substrates during the ubiquitination process (11). Moreover, in the absence of lysine residues, the substrates can be ubiquitinated in a cysteine-dependent manner (12). Therefore ubiquitin has many potential post-translational modification sites (13). Ubiquitin has a compact structure and comprises a short 3_10_-helix, a 3.5 turn of α-helix, and a β-sheet containing five strands and seven reverse circles (14). The β-sheet of the ubiquitin is a hydrophobic patch layer that includes Leu8, Ile44 and Val70 (15). The other structural centers of ubiquitin are located in Ile36, homologous to the C-terminal of E6-AP E3(HETC) and are recognized by the presence of the ubiquitin binding domain(UBD) and as well as the DUBs (16–18). Ubiquitin has eight ubiquitination sites in which seven lysine residues(K6、K11、K27、K29、K33、K48、K63) are used as receptor sites for the polyubiquitin chain formation enabling the attachment to different substrates to complete the ubiquitination process (19–21). When ubiquitin is connected to the N-terminus of the second ubiquitin, an eighth chain type, named Met1 or the “linear” chains, is created (21). Due to the complexity of ubiquitination, different Ub lysine linkages can form homotypic (linked by a single residue), heterotypic, or branched chains (22, 23). Proteomic studies have found that all possible ubiquitin chain types may coexist in the cells (19, 24–27). The results of the different ubiquitination processes in the substrate are diverse. Monubiquitination promotes the recognition of protein, the formation of complex, or allosteric regulation (21, 28, 29). While, Lys48-linked polyubiquitin chains, which are the predominant linkage type in the cells (19, 24), can target proteins for proteasome degradation (7). Conversely, Lys63-linked polyubiquitin chains, the second-most abundant chain type, perform non-biodegradable functions such as cell signaling, intracellular transport, protein kinase activation and DNA damage responses (29, 30). Furthermore, chains linked by other residues, such as K6, K27, K33, and linear ubiquitin chains, often perform various non-degradative functions, such as selective autophagy, DNA repair, and innate immunity (31). Different links among the ubiquitin molecules can form different categories of ubiquitin chains and generate different signals in the cells for the performance of various biological functions (32–35).

The E2 enzyme, which is different from E1 and E3 enzymes, plays a key transferase role in ubiquitination. UBE2L3, also called UBcH7, is one of the many E2 ubiquitin conjugating enzymes that participate in the ubiquitination of many substrate proteins. In the current study, we review the structure of UBE2L3 and its mechanism in diseases, as well as diseases associated with UBE2L3, and discuss the related challenges.

## The Structure of UBE2L3

Deep proteomic studies on the protein copy number *in vivo* have shown that UBE2L3, with 153 amino acid residues encoded, is one of the richest E2s in mammalian cell lines (36). The current three-dimensional data of the protein have indicated that UBE2L3 is highly conservative (37). The UBE2L3 enzyme retains some of the structural characteristics of universal UBC folding and is composed of a UBC domain, with up to 35% being conserved between the different family members, to provide a platform for the combination of the E1s, E3s, and activated Ub/UBL (38). The catalytic crack in this UBE2L3 structure contains a histidine (His119) similar to the acid residue in the α3-α4 ring (D117 in UBE2D1), which is directed into the lysine of the substrate protein (24, 28, 29).

**Figure 2:**
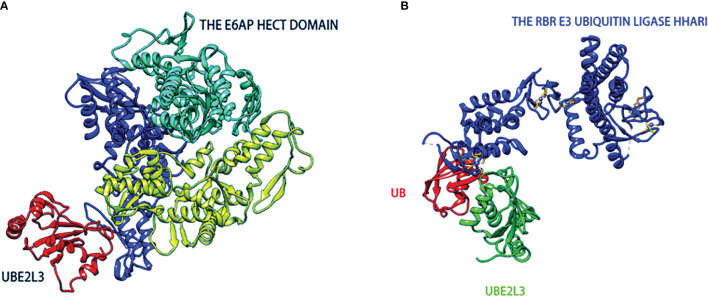
The structures of HECT-type **(A)** and RBR family **(B)** E3 ubiquitin-protein ligases in complexes with UBE2L3. **(A)** The catalytic HECT structure of E6AP is double-lobed (yellow, blue), and there is a large catalytic crack at the junction of the two lobed structures. The crack contains conserved residues(green) formed by mutated interference with the ubiquitin-thioester bond. **(B)** Ring-in-between-ring (RBR) ubiquitin E3 ligase(blue) binds to Ub(red)-UBE2L3(green) through RING/HECT hybridization mechanism.

Normally, while most E2-Ub complexes transfer Ub to the RING E3 ubiquitin ligases, UBE2L3-Ub transfers Ub to HECTs because it lacks any inherent E3-independent lysine reactivity (39). Early biochemical and structural studies have also demonstrated that UBE2L3 plays a biological role in conjunction mainly with HECT E3 (40–42). A landmark study compared the E2-Ub reactivity curve independent of E3 and found that UBE2L3-Ub was sensitive only to the thiol (cysteine) receptors (43). Furthermore, the inherent capability of UBE2L3 was not affected by the RING E3s in the ubiquitin chain. Analysis of the UBE2L3 surface revealed the presence of hot-spot residues on UBE2L3, including Lys9 in the α1, and Glu93, Lys96, and Lys100 in the 3_10_-α2 circle, as well as Phe63 in the β2-β3 circle, which contributes to the reaction of UBE2L3 with HECT E3s and the subsequent ubiquitination (44, 45). Since UBE2L3 is inherently catalytic, which means it can only interact with certain parts of E3, such as HECT’s E3 ligase and a special class of RBR E3 (43, 44). In **Figure 2A**, the structure of the E6AP Hect domain and its complex with UBE2L3 provides a preliminary view of the UBE2L3 and E2-E3 complex (46). Another study reported on the crystal structure of the RBR E3 (HHARI) and UBE2L3-Ub complexes, revealing the molecular basis of the specificity of the homologous E2/RBR E3 pairs (47), as shown in **Figure 2B**. UBE2L3 can also be associated with the disease-related E3 ligase LUBAC to form high-yield E2-E3 pairs *in vitro (* 48). The LUBAC (Linear ubiquitin chain Assembly Complex), which belongs to the RBR E3 enzyme, binds to UBE2L3 to form a specific linear ubiquitin chain linked by MET1 *in vitro*. LUBAC is composed of HOIL-1L-interacting protein (HOIP), Sharpin (SHANK-associated RH domain interaction protein in postsynaptic density) and heme-oxidized IRP2 ubiquitin ligase-1 (HOIL-1L), of which HOIP is identified as the key catalytic site of LUBAC (49). In recent years, emerging evidence has confirmed that LUBAC is a targeted part of the typical NF-κB signaling pathway that is critical for inflammation and immune development (50, 51). When stimulated by pro-inflammatory signals, the key substrates of LUBAC, IKK (NEMO), RIPK1, RIPK2, IRAKs, MyD88 and ASC (50, 52, 53) attach to the linear Ub chains through LUBAC (54). The loss of LUBAC results in impaired biological functions, including the attenuation of NF-KB and mitogen-activated protein kinase (MAPK)-mediated signaling pathways and increased cell death (54). In summary, UBE2L3 reacts mainly with HECT or RBR (RING-in-between-RING) E3 ligase on the ubiquitin chain. The E3 ubiquitin ligases linked to UBE2L3 and their corresponding functions are summarized in **Table 1** (40, 46, 48, 55–72). In the current study, we review the structure of UBE2L3, and its mechanism in diseases, as well as diseases related to UBE2L3 and discuss the related challenges.

**Table 1:** The E3 Ubiquitin ligases linked to UBE2L3 and their related functions.

E3 Ubiquitin ligases	Functions	References
E6AP	Used by HPV-16 E6 protein to ubiquitinate p53/TP53.	(40)
OspG	Combines with UBE2L3-Ub to inhibit the activation of NF-κB.	(46)
sopA	Interferes with the host’s ubiquitination pathway.	(55, 56)
NleL	Interacts with UBE2L3 to provide a framework for bacterial pathogenesis.	(57)
NDFIP1	Enhances the ITCH-mediated ubiquitination of MAP3K7 by recruiting UBE2L3 to ITCH.	(58)
PARKIN	Involved in the ubiquitination and degradation of misfolded proteins.	(59, 60)
ARIH1	Catalyzes the ubiquitination of target proteins together with UBE2L3.	(61)
ARIH2	Synthesis of the ubiquitin chain by combining UBE2L3 and the ubiquitin protein.	(62)
RNF19A	Binds with UBE2L3 through the RING-finger/IBR domain, localized in the centrosome and probably functions in the microtubule organizing centers.	(63)
RNF19B	Degrades urokinase-1 and affects the function of natural killer cells.	(64)
RNF144B	Involves in ubiquitination and degradation of p21 and switching a cell from p53-mediated growth arrest to apoptosis.	(65)
RNF31	Binds to UBE2L3 to specifically form a linear ubiquitin chain linked by MET1 *in vitro*.	(48)
CCNB1IP1	Regulates the level of cyclin-B and involves in the regulation of cell cycle progression *via* the G2 phase.	(66)
CBL	Identifies activated receptor tyrosine kinases and promote the ubiquitination by UBE2L3 and terminates signaling.	(67)
TRAF6	Ubiquitinates neurotrophic factor receptor interaction factors and guides nuclear localization through the K63 chain.	(68)
BRCA1/BARD1	Does not catalyze its ubiquitinating.	(69)
RNF213	Has not been reported.	(70)
TRIM63	Involves in the degradation of myofibrillar actin and myosin.	(71)
TAX	Synthesis of the mixed-linked polyubiquitin chain that can directly activate IKK.	(72)

## Review and Discussion

### The Signaling Pathways Associated With UBE2L3

As a ubiquitin conjugating E2 enzyme, UBE2L3 can work with E1s and E3s to catalyze multiple substrate proteins to complete the ubiquitination process. A large number of reports have indicated that UBE2L3 is involved in the occurrence and development of tumors, immune diseases, and Parkinson’s disease, through mainly the following signaling pathways: The nuclear transcription factor-kappa B (NF-κB) signaling pathway, GSK3β/p65 signaling pathway, p53 signaling pathway, autophagy mediated by p62, and DSB repair pathway.

### The NF-κB Signaling Pathway

NF-κB includes a number of transcription factors involved in regulating biological responses and it regulates a variety of cellular functions in the immune system, including cell survival, differentiation, and proliferation (73). Ubiquitination has an essential effect on the regulation of NF-κB signaling pathways. Under normal conditions, NF-κB, combined with an inhibitory protein of the IκB family, is sequestrated in the cytoplasm. When cells are stimulated by different types of agents, including inflammatory cytokines such as interleukin-1 (IL-1), tumor necrosis factor α (TNFα), and microbial products such as lipopolysaccharide (LPS), the IκB kinase (IKK) complex is activated. The IKK complex consists of IKKα, IKKβ, and NEMO (also called IKKγ or IKKAP). The activated IKK phosphorylates IκB and promotes the degradation of these inhibitors through the ubiquitin proteasome system, that allows the NF-κB to translocate to the nucleus and to initiate the transcription of a large number of target genes (74, 75). Some of the key components that are generated after NF-κB activation in turn regulate NF-κB activity negatively. However, ubiquitination is a highly dynamic and reversible process in which the ubiquitin chains can be removed from the substrates by DUBs (9). Several DUBs, such as CYLD (76, 77), A20 (78), and OTULIN (79), act as negative regulators to inhibit the activity of NF-κB (77).

In the field of ubiquitin regulation of NF-κB activation, the E2 ubiquitin conjugating enzymes UBE2N and UBE2D1 have been the most highly studied. UBE2N regulates NF-kB activation through the IL-1 pathway. In the IL-1 pathway, IL-1 binds with the corresponding receptors to recruit many different cellular signaling molecules, such as MyD88 and TRAF6. TRAF6 is a RING E3 ubiquitin ligase, which functions together with UBE2N to catalyze the synthesis of the K63 - polyubiquitin chain (80). This K63 polyubiquitin chain activates the TGF-β activated kinase 1 (TAK1) kinase complex, which in turn phosphorylates IKKβ leading to the activation of IKK (81), while UBE2D1 regulates the NF-kB activation through the TNFα pathway. In the TNFα pathway, TNFα binds to the corresponding receptors to recruit TRAF2, TRAF5, cIAP1, cIAP2 and RIP, and cIAP1 together with the UBE2D1 synthesized polyubiquitin chains with different linking modes, including the K63, K11 and linear ubiquitin chains. The heterogeneous polyubiquitin chains promote ubiquitin RIP, which activates the TAK1 kinase complex, leading to the activation of IKK and the phosphorylation of IKKβ (82).

**Figure 3:**
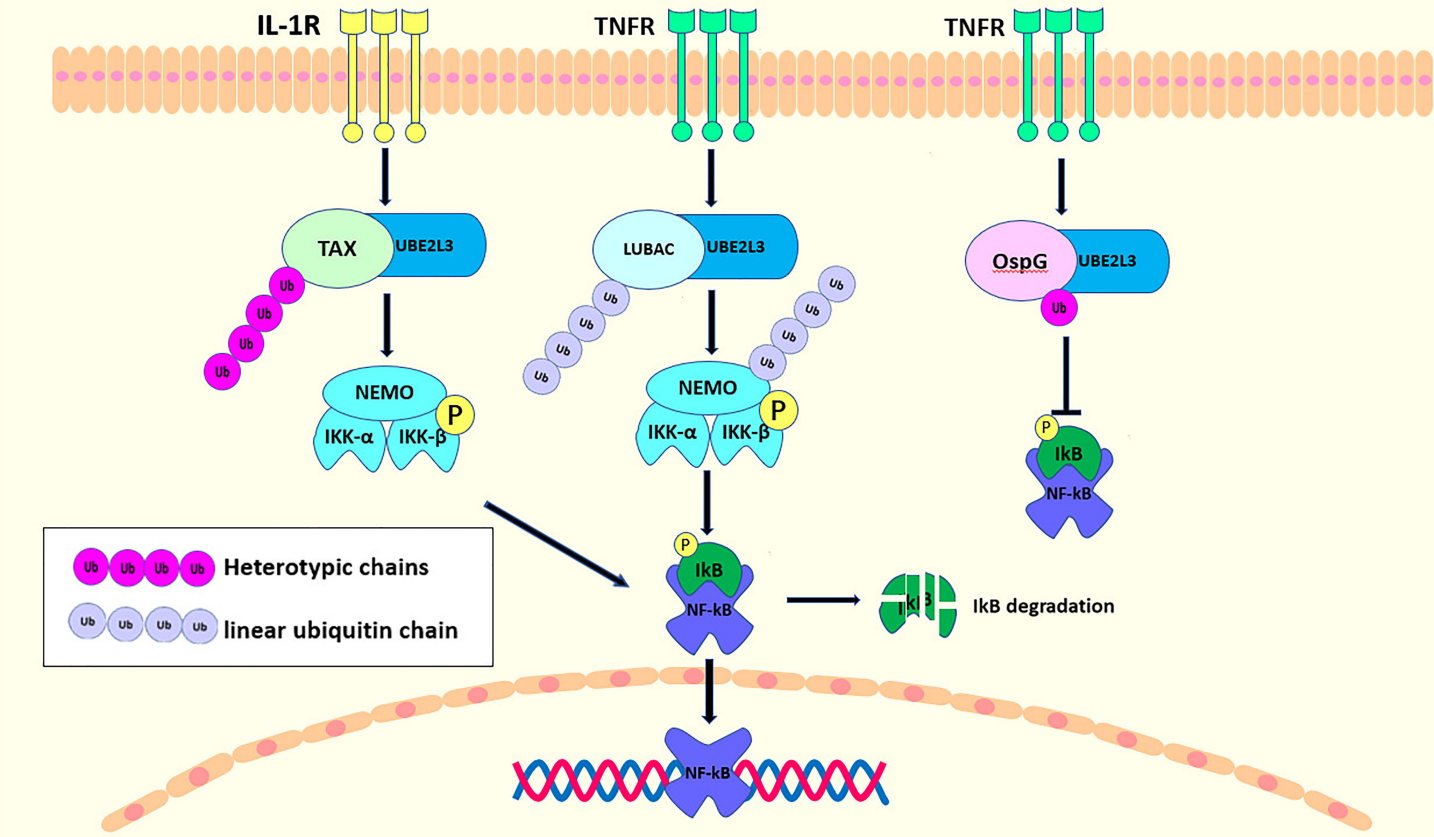
UBE2L3 binding with different E3 ubiquitin ligases acts on NF-κB signaling pathway. Upon the stimulation of IL-1R, TAX interacts with UBE2L3 to synthesize the heterotypic chains, resulting in IκB phosphorylation, and the degradation and activation of NF-κB. In the TNFR pathway, UBE2L3 interacts with LUBAC to synthesize first, a linear ubiquitin chain, then there is a linear ubiquitination of NEMO and a phosphorylation of IKKβ, ultimately leading to the activation of NF-κB. While OspG can interact with UBE2L3-Ub conjugates to improve the kinase activity of OspG, which inhibit the activation of NF-κB.

UBE2L3, an E2 ubiquitin conjugating enzyme, is also involved in the regulation of the NF-κB signaling pathway. Current studies have indicated that there are many, although controversial mechanisms through which UBE2L3 regulates NF-κB activation. Tax, a transactivator encoded by human T-cell leukemia virus Type 1 (HTLV-1), is critical in the life cycle of the virus (83). UBE2L3 combines with Tax, a novel E3 ubiquitin ligase, for the assembly of the mixed-linked polyubiquitin chain in a variety of linkages, although not for the K63-polyubiquitin chain, which activates IKK directly (72). UBE2L3, together with Tax, can synthesize the mixed-linked polyubiquitin chain that activate IKK directly. However, it cannot activate the TKA1 complex, as this can be activated only by the K63-polyubiquitin chain (84). However, UBE2L3 linked to different E3 ubiquitin ligases play different roles in NF-κB regulation. OspG kinase, one of the host invasion and cell-disrupting shigella effectors, inhibits the NF-κB pathway activation by blocking phospho-IκBα degradation (85). OspG can only interact with several E2-conjugating enzymes that contain Ub (85). A structural and biochemical study showed that OspG binds preferentially to the UBE2L3-Ub conjugate (46). First, the stable UBE2L3-Ub complex is formed by the disulfide bond between the catalytic cysteine (C86) in UBE2L3 and the C-terminus of Ub (G76C) (86). The ubiquitin is located in the “open” conformation of UBE2L3 and interacts with the C-terminus of OspG *via* its I44 patch. UBE2L3 then binds to OspG *via* the two conserved rings that are necessary for the E3 ligase recruitment. Although UBE2L3-Ub binds to OspG at sites far from the active kinase site, it increases its kinase activity and inhibits NF-κB activation (46). UBE2L3 is also the main E2 conjugating enzyme linked to LUBAC to regulate NF-κB activity. When TNFα binds to its receptor to recruit the LUBAC, UBE2L3 binds to HOIL-1L in LUBAC to promote the linear ubiquitination of NEMO, which in turn recruits the TNF signaling complex. NEMO contains two ubiquitin binding sites that not only cross-link and stabilize other ubiquitin components associated with the activated TNFR1 complex, but also acts as an adaptor of IKKα/IKKβ kinase. IKKs are recruited into the TNFR1 complex and activated, then the activated IKKs phosphorylates IκBα to promote the activity of NF-κB (20). UBE2L3 interacts with the three E3 ligases to regulate NF-κB activity, as shown in **Figure 3**. According to additional studies on the regulation of UBE2L3 in NF-κB, a UBE2L3 inhibitor has been found. Dimethyl fumarate (DMF), a derivative of fumaric acid, has neuroprotective and immunomodulatory effects (87–89). TLR7 stimulation leads to intracellular accumulation of the linear ubiquitin chains, similar to TNFα. Co-overexpression of UBE2L3 and LUBAC enhanced NF-KB activation by TLR7-driven, and DMF as an antagonist of UBE2L3 inhibited the response to TLR7 activation. Unfortunately, these effects of DMF on UBE2L3 were only published in the conference abstract FRI0271 without relevant research articles, so the specific mechanism of DMF on UBE2L3 is not very clear.

Additional studies have shown that UBE2L3 regulates NF-κB by binding with different E3 ubiquitin ligases to form different types of ubiquitin chains, leading to positive or negative regulatory reactions. Therefore, future research should pay more attention to the structure and biochemical mechanisms of UBE2L3 that link the different E3 ligases. This will provide a theoretical basis for the discovery of additional antagonists.

### GSK3β/p65 Signaling Pathway

Glycogen synthase kinase 3β (GSK3β) is a serine-threonine kinase that phosphorylates and inactivates glycogen synthase (90). GSK3β participates in a variety of signaling pathways to regulate a plethora of cellular activities, such as metabolic activities, transcriptional regulation, neuronal functions, vesicle transport, cell cycles, as well as tumorigenesis and tumor development (91). GSK3β upregulates the activity of NF-κB, and the enhanced activity of NF-κB further stimulates and participates in the pathways of cell proliferation, the production of the tumor promoting cytokines and the promotion of anti-apoptosis (92–94). The inhibition of GSK3β enhances the activity of transcription factors that promote epithelial to mesenchymal transformation (94, 95).

**Figure 4:**
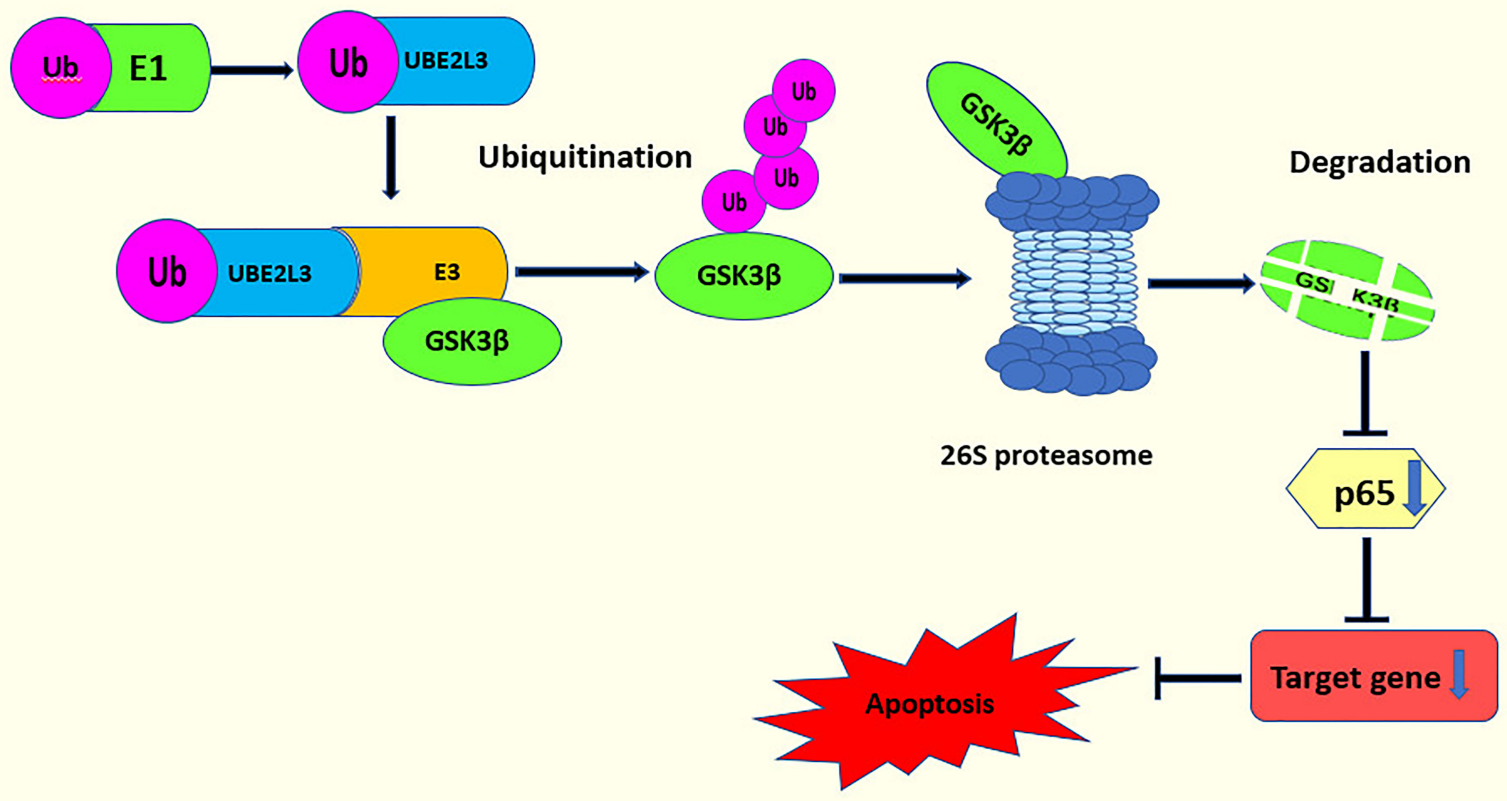
UBE2L3 acts on the GSK3β/p65 signaling pathway. UBE2L3 participates in the ubiquitination of GSK3β and promotes the degradation of GSK3β, leading to a decrease on the expression of p65, thereby inhibiting apoptosis.

Studies have shown that the knockdown of UBE2L3 and the overexpression of GSK3β can increase NF-κB activity. Moreover, further research has demonstrated that there is a negative feedback regulation between UBE2L3 and GSK3β showing an interaction between UBE2L3 and GSK3β. p65 is the most important subunit and active part of NF-κB. The knockdown of GSK3β can reduce the expression of p65, inhibit the phosphorylation of p65, and further inhibit NF-κB activity. Therefore, the knockdown of UBE2L3 promotes the expression of GSK3β, which activates p65 and leads to NF-κB activation (96) (**Figure 4**). In summary, UBE2L3 regulates NF-κB activity through the GSK3β/p65 signaling pathway. In this process, in order to clarify whether the degradation of GSK3β by UBE2L3 depends on the ubiquitin-proteasome system, the protein expression level of GSK3β significantly decreases when UBE2L3 is overexpressed, while the protein level of GSK3β significantly recovers when MG132, a proteasome inhibitor, is added. This suggests that the degradation of GSK3β by UBE2L3 depends on the ubiquitin proteasome pathway (96); however, the specific mechanism of GSK3β degradation by UBE2L3 was not clarified in this study. Therefore, further studies should evaluate which of the E3 ubiquitin ligases UBE2L3 interacts with to complete the ubiquitination of GSK3β and which ubiquitin chains are formed during the ubiquitination of GSK3β.

### DNA Double Strand Break (DSB) Repair

Organisms depend on genetic code to perform various cellular functions, so DNA damage poses a threat to organisms. In the face of DNA damage, specialized DNA repair mechanisms can maintain genome integrity (97). In eukaryotic cells, the most destructive type of DNA damage is DSB damage, which may be repaired by two primary, mechanically different pathways: homologous recombination (HR) and non-homologous terminal joining (NHEJ) (97). HR replaces the missing or damaged DNA sequences in a high-fidelity manner that depends mainly on the intact sister chromatid as a template. Therefore, HR only functions in the S and G2 phases of the cell cycle in which a sister chromatid is present (97, 98). By comparison, NHEJ remains active during the whole cell cycle, repairing DSB by connecting directly to the ends of chromosomes without using sister chromatids as templates. However, because of the way NHEJ works, it is prone to errors in the repair process (97, 99). The failure of DSB repair pathways not only affects the stability of the genome but also leads to the development of cancer and resistance to anticancer therapy (100).

UBE2L3 is involved in regulating DSB repair through different mechanisms. BRCA1, a tumor suppressor protein of breast and ovarian cancer, binds to a RING finger protein BARD1 to form a ring heterodimer (69) and it functions as an E3 ubiquitin ligase for ubiquitination (101). BRAC1 is involved in several processes, including DSB repair, cell cycle progression and transcription. It has been reported that BRCA1 undergoes autoubiquitination and interacts with a phosphorylated histone 2AX (pH2AX), in an early cellular response induced by DSB (102). Several E2 ubiquitin conjugating enzymes in BRCA1 that depend on ligase reactions were screened out, including UBE2H, UBE2D2, UBE2D3, UBE2R1, and UBE2L3 (103); however, only UBE2D3 and UBE2L3 combine with BRCA1 (69). In contrast, the complex formed by UBE2D3 binding with BRCA1 catalyzes the ubiquitination of H2A/H2AX (104, 105), while UBE2L3 was inactive in the Ub ligase activity test (69). Therefore, the specific mechanism of UBE2L3 binding to BRAC1 without catalytic ubiquitination needs to be studied further.

**Figure 5:**
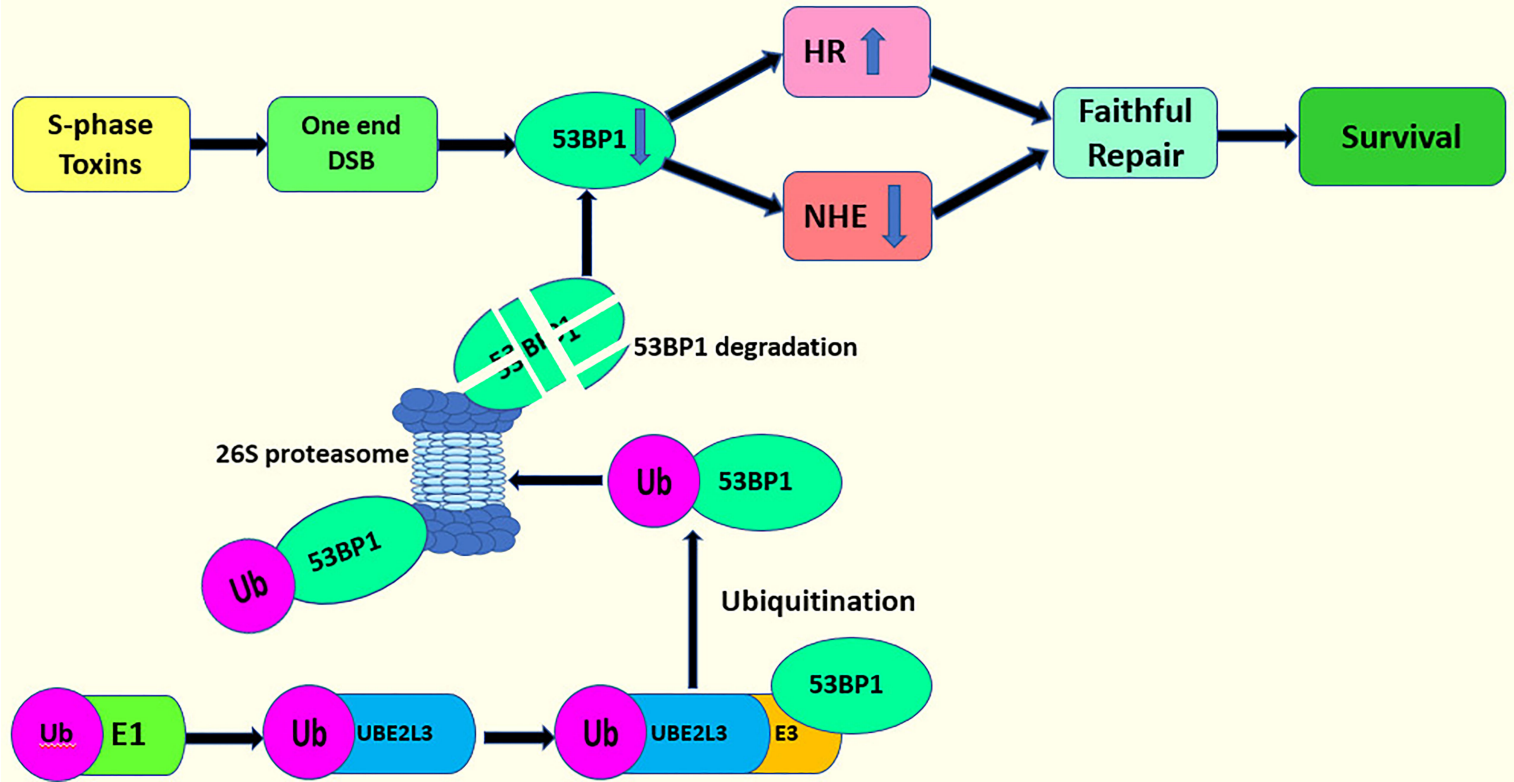
UBE2L3 acts on DSB repair and cell survival.

Another important regulator in DSB repair is the tumor suppressor protein p53 binding protein 1 (53BP1) (100). 53BP1, combined with Rif1, co-inhibit BRCA1-dependent HR, thereby facilitating NHEJ in the G1 phase (106, 107). Conversely, 53BP1/Rif1, antagonized by BRCA1, favors HR in the S and G2 phases (108, 109). UBE2L3 regulates the protein stability of 53BP1 through ubiquitination to determine DSB repair mode. The deletion of UBE2L3 stabilizes the protein level of 53BP1, causing the cells to choose NHEJ over HR in the repair of DSB, which can threaten the DNA-damaged cells. These DNA-damaged cells create obstacles in the process of creating the DNA replication fork, leading to the breakdown of the DNA replication fork and the generation of a one-ended DSB (100). This one-ended DSB requires repair by HR rather than by NHEJ (100, 109). A one-ended DSB repaired by NHEJ results in radial chromosomes and cell death (100, 110–112). Therefore, the overexpression of UBE2L3 downregulate the protein levels of 53BP1. Then, the cells choose HR to repair the one-ended DSB (100) (**Figure 5**). However, whether UBE2L3 regulates 53BP1 during DSB repair through the ubiquitin proteasome system has not been clearly stated, so further studies should determine this point.

### p62 Signaling Pathway

The ubiquitin–proteasome system (UPS) and autophagy are two different interlinked pathways, which play a key role in cellular proteostasis under normal and stressed conditions. In eukaryotes, two interconnected pathways remove misfolded proteins (113). In UPS, the misfolded proteins are linked to the polyubiquitin chains *via* lysine residues to form a ubiquitin-protein complex. Ubiquitinated proteins are recognized by the proteasome, deubiquitinated by DUB, and ultimately degraded (114). However, when the UPS is damaged or the misfolded proteins exceed its repair capacity, these proteins can be stored to create larger structures that then serve as substrates for autophagy (113, 115). This procedure is also referred to as aggregation. During aggregation, the double-membraned organelles known as autophagosomes insulate the protein substrates (116). After the fusion of the autophagosome and lysosome, the substrate proteins degrade (116).

**Figure 6:**
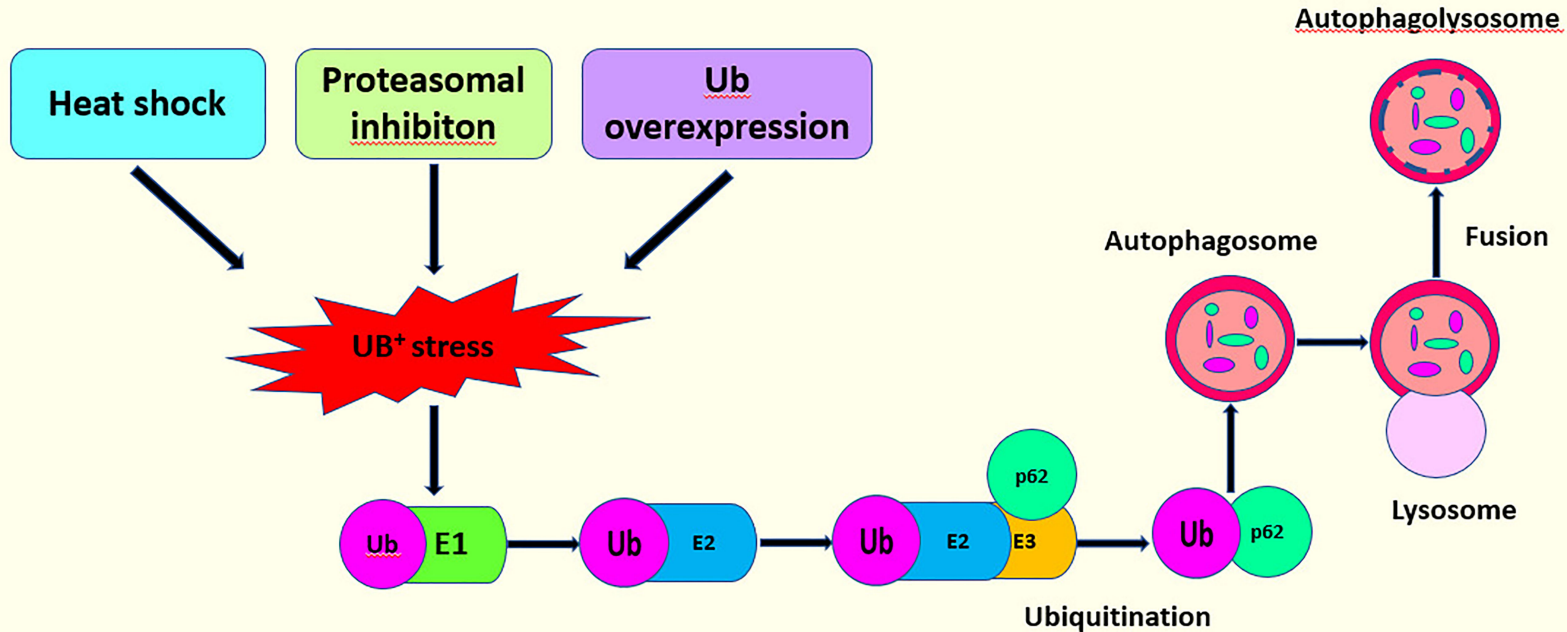
This model describes the interaction of the p62 signaling pathway with the UPS to activate autophagy.

p62 is an autophagy receptor that was discovered in mammals (117). Shin named it Sequestosome 1 (SQSTM 1) because it can create aggregates (118). p62 links the UPS and autophagy (113, 119). Under conditions of stress such as heat shock, the overexpression of ubiquitin and the inhibition of proteasome, the amount of ubiquitin increases abnormally, causing UB+ stress and further inducing p62 ubiquitination. A large number of ubiquitinated proteins exceed the capacity of the UPS, and the body finally unlocks the autophagy pathway to degrade the ubiquitinated proteins (6, 96, 120) (**Figure 6**). In a previous report, UBE2L3 interacted with Parkin (60). Parkin consists of two domains with different functions: a C-terminal ring-box domain and an N-terminal Ubl domain (60). UBE2L3 can bind to the C-terminal ring-box of Parkin to specifically discharge ubiquitin into the active sites of Parkin (43). In another study, however, a set of UBE2 enzyme may regulate Parkin-mediated autophagy by p62 signaling pathway. The autophagy clearance of depolarized mitochondria can be reduced significantly by inhibiting the E2 enzymes, UBE2L3, UBE2N, or UBE2D2 and UBE2D3 (UBE2D2/3). However, after single UBE2 knockdowns, polyubiquitin and p62 were still found in mitochondria indicating that UBE2N, UBE2L3, and UBE2D2/3 participate cooperatively in the Parkin-mediated autophagy *via* the p62 signaling pathway (121). Further, another study confirmed that UBE2L3, UBE2D and UBE2N were positive regulators of Parkin mitochondrial translocation. However, this study confirmed that the knockout of UBE2L3, UBE2D, and UBE2N alone could delay Parkin translocation to and p62 recruitment by damaged mitochondria. The double knockout experiment showed that the combined silencing UBE2L3 and UBE2D had an additive effect, which confirmed the redundancy of UBE2D and UBE2L3. However, the combined knockout of UBE2N with UBE2D or UBE2L3 showed no strong additive effect, indicating that UBE2N, UBE2D, and UBE2L3 were not redundant, but acted in different stages. Moreover, although these three E2s catalyzed the formation of the ubiquitin modified Parkin *in vitro*, only UBE2L3 promoted the obvious self-ubiquitination of Parkin (122).

### p53 Signaling Pathway

p53 is a protein that inhibits tumor growth and plays an important role in tumor growth by DNA replication, controlling cell cycle and uncontrolled cell division (123). p53 is formed from 393 amino acids, and has several primary functional domains: DNA binding, transcription, regulatory domain and tetramerization (124). The structure of this protein comprises a loop helical structure (L, S, H) and five conserved regions (I, II, III, IV, V). The five conserved domains overlap with the loop helix domains, and together, they form part of the protein’s three-dimensional structure. Furthermore, abnormalities in the three-dimensional domain of p53 may lead to mutations (124).

Although p53 has many biological functions, its expression levels are low in unstressed cells due to the strict control of the ubiquitin-proteasome degradation system (125). Several ubiquitin ligases, such as E6AP (40), ARF-BP1 (126), MDM2 (127), COP1 (126), and PIRH2 (126, 128), can bind to p53 and lead to its degradation, in which EA6P plays a leading role. E6 oncoprotein, derived from HPV, promotes the development of cervical cancer by degrading p53 (129). Furthermore, several ubiquitin-conjugating enzymes, including UBE2D1, UBE2E1, UBE2L3, UBE2D2, and UBE2D3 are involved in the ubiquitination of p53. However, only UBE2D1 and UBE2L3 can interact with E6AP in the ubiquitination of p53, indicating that UBE2D1 and UBE2L3 are E6AP ubiquitin ligase E2s (130, 131). The aromatic hydrocarbon receptor, a ligand-activated receptor, acts as a regulator of p53 and promotes the ubiquitination and degradation of p53 by controlling the expression of the UBE2L3 protein (132). However, further studies are required to determine whether there are other E3s and regulators involved in the interactions between UBE2L3 and p53 in the ubiquitin proteasome system.

### Relevance of UBE2L3 to Disease

The localization of UBE2L3, in Chromosome (Chr) 22q11.2-13.1 (133), is unusual for E2 ubiquitin conjugating enzymes, because it has no direct homolog in yeast, which limits the exploration of its biological functions. UBE2L3 has important mammalian functions since the decrease of this enzyme in mice leads to growth retardation and placental vascular defects causing prenatal or perinatal death (134). The expression levels and activation statuses of UBE2L3 alter during cell differentiation and in the cell cycle. The downregulation expression of UBE2L3 prolongs the proportion of cells in the S phase while the upregulation expression of UBE2L3 increases the proportion of cells in the G1 phase relative to the S phase (135, 136). UBE2L3 also acts in several inflammatory disease (137–139) and the formation and progression of tumors (96, 100, 140). Therefore, this review summarizes the role of UBE2L3 in different types of diseases.

### UBE2L3 and Immune Disorders

The immune system is important for self-protection. It enables the body to remove antigens for the purpose of self-protection (141). Immunodeficiencies can lead to a variety of viral and bacterial infections, while over immunity always results in autoimmune diseases (142). The NF-κB signaling pathway regulates the expression of immune-related genes extensively, and the role of UBE2L3 in immune-related diseases relies mainly on the NF-κB pathway (143). It has been reported that in addition to the NF-κB signaling pathway, UBE2L3 regulates immunity through several other pathways.

In genome-wide association studies (GWAS) and other genetic studies, UBE2L3 has been to be implicated in a variety of autoimmune diseases (**Table 2**) (139, 144–158). A few of the single nucleotide polymorphisms (SNPs) depict risk alleles related to several autoimmune diseases, including systemic lupus erythematosus(SLE) (145, 146, 148), Crohn’s disease (155), rheumatoid arthritis(RA) (150), inflammatory bowel disease(IBD) (153), celiac disease(CD) (158), psoriasis (156), diffuse systemic skin sclerosis (dcSSc) (159), and juvenile idiopathic arthritis(JIA) (152). Moreover, the risk allele rs5754217, located on UBE2L3, is related significantly to SLE and RA, indicating that UBE2L3 may be a common susceptibility site for these two autoimmune rheumatic diseases (137). A haplotype analysis has shown that the structure of UBE2L3 loci is relatively simple, and two haplotypes may extend to the whole gene length and cover most of the genetic variations. A recent study also showed that a single haplotype block, which is related to multiple other autoimmune diseases, is relevant to SLE (147). UBE2L3 was associated recently with the GWAS of chronic hepatitis B in the Han community (157), showing that UBE2L3 is required for overcoming hepatitis B virus infections. In the original GWAS and in a combined analysis of two independent Dutch replication cohorts, two novel potential risk genes for Crohn’s disease were identified: UBE2L3 and BCL3. The high expression of the UBE2L3 gene was related closely to the risk allele(rs2298428), located on the UBE2L3 gene. The overexpression of UBE2L3 results in a faster degradation of the NF-κB precursor and reduced NF-κB production, further inhibiting the innate immune response (139). TNFAIP3 and TNIP1, which are ubiquitin-related genes, have been shown to be correlated with SLE, RA, and systemic sclerosis (SSc). Moreover, diffuse cutaneous systemic sclerosis(dcSSc) has been reported to have a strong association with these two genes (159–161). This is supported by a case-control study on the genetic background of dcSSc in Japanese that showed that UBE2L3 may also be involved in the genetic background of SSc through ubiquitination, and that this mechanism may act on the pathogenesis of dcSSc (159). Due to limited understanding of the overall genetic structure of juvenile idiopathic arthritis, relatively few loci associated with the disease have been identified (162). In addition, an immunomicroarray sequence analysis, the association of JIA with six loci, which were C5orf56IRF1(rs4705862), RUNX1 (rs9979383), RUNX3(rs4648881), ERAP2LNPEP(rs27290), PRR5L(rs4755450), and UBE2L3(rs2266959), was confirmed (163). Using an analysis of expression quantitative trait loci, a large number SNPs of UBE2L3 was associated with disease risks relevant to the overexpression of UBE2L3. Therefore, a higher expression and higher activity of UBE2L3 may be the cause of autoimmune diseases caused by altered immune response pathways. Furthermore, the specific mechanism of UBE2L3 in autoimmune diseases needs to be studied further.

**Table 2:** Genetic studies showing an association between UBE2L3 and autoimmune and infectious diseases.

Disease	SNP	A1/A2	OR (95％CI)	P	Reference
**SLE**	rs2298428	T/C	1.29 (1.18-1.41)	2.4E-08	(144)
	rs463426	C/TThe Shigella Flexneri Effe	0.89 (0.79-1.02)	2.50E-05	(144)
	rs5754217	T/G	1.20 (1.13-1.27)	2.30E-06	(145)
	rs131654	G/T	0.78 (0.74-0.83)	2.99E-16	(146)
	rs140490	G/C	1.30 (1.21-1.39)	8.62E-14	(147)
	rs7444	T/C	1.26 (1.13-1.41)	2.21E-14	(148)
**RA**	rs2298428	G/A	1.09	2.50E-10	(149)
	rs5754217	G/T	1.10	4.80E-05	(150)
	rs11089637	T/C	1.08 (1.05-1.11)	2.10E-09	(151)
**JIA**	rs2266959	T/G	1.24 (1.15-1.33)	6.20E-09	(152)
**IBD**	rs2266959	T/G	1.11 (1.07-1.15)	1.39E-16	(153)
**UC**	rs5754217	T/G	1.44 (1.20-1.73)	7.69E-05	(154)
**Crohn’s disease**	rs2298428	A/G	0.97	7.71E-04	(139)
	rs181359	G/A	1.10 (1.06-1.15)	4.80E-16	(155)
**Celiac disease**	rs2298428	T/C	1.13 (1.08-1.19)	1.84E-07	(144)
**Psoriasis**	rs4821124	T/C	1.37 (1.21-1.54)	4.72E-07	(156)
**Hepatitis B**	rs4821116	G/A	0.82 (0.77-0.87)	1.71E-12	(157)

SLE, Systemic lupus erythematosus; RA, Rheumatoid arthritis; JIA, Juvenile Idiopathic Arthritis; IBD, Inflammatory bowel disease; UC, Ulcerative Colitis; SNP, Single-nucleotide polymorphism. A1/A2 indicates the minor allele/major allele of the SNP.

### UBE2L3 and Cancer

In recent years, an increasing number of studies have shown the correlation between UBE2L3 and tumors. Studies have found an abnormal expression of UBE2L3 in the cell lines and tumor tissues from many patients with a tumor indicating that UBE2L3 is involved in a number of cancer-related signaling pathways.

GSK3β reportedly stimulates the development of certain tumors, including those in pancreatic cancer, colorectal cancer and myeloma cancer, while inhibiting the development of other tumors, including those in lung, esophageal, and breast cancer (164–166). A high level of UBE2L3 in hepatocellular carcinoma inhibits p65 by down-regulating GSK3β through the ubiquitin-mediated proteasome degradation, rather than by the classical NF-κB signaling pathway, suggesting that an overexpression of UBE2L3 in hepatocellular carcinoma promotes apoptosis evasion through the inhibition of the GSK3β/p65 pathway (96).

The p53 pathway plays an important role in a many of the biological functions of the body, including cell cycle regulation, metabolism, aging and development, reproduction, and the inhibition of tumor expression (167–169), and has been considered as a crucial anticancer target (170). The E6 oncoprotein derived from HPV promotes the growth of cervical cancer by degrading p53 and blocking the p53-mediated growth arrest and apoptosis (129, 171). This relied mainly on UBE2L3 and E6AP (129, 172–174). Other studies have shown that UBE2L3 may reduce HeLa cell apoptosis by stimulating ubiquitination and the degradation of p53 (132, 175).

Abnormalities in the DSB repair pathway result in the growth of tumors and resistance to antineoplastic treatment (100). The formation of the tumor suppressor signal 53BP1 foci plays a role in selecting the DSB repair pathway. Controlling the level of 53BP1 showed an impact almost as powerful as lesion formation in DSB repair and in establishing cell sensitivity to anticancer therapies (176). A study that analyzed protein expression levels of UBE2L3 and 53BP1 in several breast cell lines showed a negative correlation between the two proteins, particularly in triple-negative breast cancer cells. All of these data reveal that the UBE2L3/53BP1 axis may act on the occurrence and development of tumors. Through further research, the regulation of UBE2L3 can stabilize the levels of 53BP1 protein, resulting in reduced HRs for DSB repair (100).

UBE2L3 controls the protein stability of several signaling molecules, which acts on cancer regulation. A study reported that UBE2L3 promotes the ubiquitination and degradation of p27kip1 in non-small-cell lung cancer (NSCLC), and further study suggested that high expression of UBE2L3 led to the down-regulation of p27KIP1 protein levels, which are relevant to a poor prognosis for NSCLC (177). Another study identified the oncogenic UBE2L3-KRAS fusion in DU145 prostate cancer cells using an integrative genomics approach, and further investigation demonstrated that the fusion of UBE2L3 with KRAS may lead to the progression of metastases in rare subsets of prostate cancer (140).

### UBE2L3 and Parkinson’s Disease

Parkinson’s disease (PD) is a common neurodegenerative disease with complex clinical features. Genotypic and linkage analyses have shown that the most common cause of sporadic early and late PD cases was mutations in the PARK2 gene encoding Parkin which act as an RBR E3 ligase (178, 179). Parkin may have a neuroprotective function by regulating mitochondrial autophagy. The N-terminal UBL domain of Parkin can inhibit its activity automatically; thus, the interaction between the E2-loaded ubiquitin and Parkin is affected (180–183). When Parkin’s autoinhibitory state is removed, the dual PINK1 kinase-mediated phosphorylation activates Parkin (184–188). The activated Parkin can then interact with the various E2-loaded ubiquitin, including UBE2L3, to induce a process of mitochondrial autophagy. Studies have suggested that UBE2L3 may act on Parkin-dependent autophagy through the p62 signaling pathway (121, 122). However, there remains controversy with respect to the interactions between Parkin and UBE2L3, UBE2N and UBE2D for the induction of mitochondrial autophagy, which was described in the section on the p62 signaling pathway. This detailed mechanism should be studied further.

## Future Perspectives and Conclusions

Ubiquitination is an important post-translational modification that regulates the various biological functions of cells by modifying proteins. In recent years, an increasing number of researchers have begun paying attention to ubiquitination due to an increase in studies on tumor progression and treatment, and in order to seek effective antineoplastic treatment (189). UBE2L3 is an E2 conjugating enzyme that exists widely in eukaryotes. It plays a critical role in the UPS. UBE2L3 is expressed abnormally in several human cancers, including hepatocellular carcinoma, NSCLC and cervical cancer. UBE2L3 can stimulate the occurrence, development, and metastasis of human tumors by regulating different signaling pathways relevant to human tumors and a variety of proteins that do not participate in the above signaling pathways. p53 can promote tumor cell apoptosis and is an important tumor suppressor. Inhibiting UBE2L3 can activate p53, which may provide a direction for the development of new anticancer drugs. However, the regulation of UBE2L3 on GSK3β/p65, DSB repair and other signaling pathways also requires further study, and the specific mechanisms of UBE2L3 in other tumors and their potential as novel antineoplastic treatment should be investigated. Although the findings of previous studies, have shown that UBE2L3 is a promising target for therapies for immune diseases and Parkinson’s disease, the specific molecular mechanism of the involvement of UBE2L3 in regulating immune diseases and Parkinson’s disease should be studied further. We recommend that future studies focus on the development and evaluation of UBE2L3 inhibitors and demonstrate their efficacy and safety in the treatment of these diseases.

## Author Contributions

XZ: Writing—original draft preparation, investigation, and figure preparation. CH: Investigation and figure preparation. YLiu: Investigation. RS: Investigation. YZ: Investigation. YLi: Conceptualization, methodology, and supervision. All authors contributed to the article and approved the submitted version.

## Funding

This work was supported by Fundamental Research Funds from the Central Universities (lzujbky-2021-ct18), the Development and Reform Commission Project of Gansu Province (2020-2022), the Education Department of Gansu Province (2021jyjbgs) and the Major Science and Technology Special Project of Gansu Province (20ZD7FA003).

## Conflict of Interest

The authors declare that the research was conducted in the absence of any commercial or financial relationships that could be construed as a potential conflict of interest.

## Publisher’s Note

All claims expressed in this article are solely those of the authors and do not necessarily represent those of their affiliated organizations, or those of the publisher, the editors and the reviewers. Any product that may be evaluated in this article, or claim that may be made by its manufacturer, is not guaranteed or endorsed by the publisher.
